# Optimizing TrueBeam jaw calibrations to reduce positional errors at customizable gantry and collimator angles

**DOI:** 10.1002/acm2.70870

**Published:** 2026-10-22

**Authors:** Caleb M Sample, Marcus Sonier, Parmveer Atwal

**Affiliations:** ^1^ Department of Medical Physics Royal Victoria Regional Health Centre Barrie Ontario Canada; ^2^ Department of Medical Physics BC Cancer ‐ Abbotsford Abbotsford British Columbia Canada

**Keywords:** collimator, gantry sag, jaw calibration, junction, quality assurance jaw calibration collimator, TrueBeam

## Abstract

**Background:**

Clinical medical physicists in a radiotherapy setting are aware of the importance of maintaining linac collimators with high positional accuracy, especially when used to form junctions; however, there is a lack of quality control options available. This is especially true when trying to improve jaw matching in junctions or when trying to maintain accurate jaws across gantry angles other than 0 degrees.

**Purpose:**

In this work, we address this quality assurance niche by developing a jaw calibration optimization program, which defines jaw calibration positions to minimize jaw positional errors and junction matching errors over customizable gantry and collimator angles.

**Methods:**

A jaw calibration optimization program was developed and evaluated on two Varian TrueBeam linacs. Half beam blocked MV EPID images were acquired to characterize jaw positions and clinically relevant breast junction errors across a set of gantry and collimator angles. Collimator encoder values were correlated with measured jaw positions, allowing the jaws to be set precisely during calibration. The Jaw calibration program analyzed DICOM images, modelled encoder‐position relationships, and optimized the calibration origins for all four jaws. The jaw calibration was performed and then verified by redoing the analysis with the new jaw settings, and standard QA of graph paper jaw position, light field radiation coincidence, and jaw matching.

**Results:**

The jaw calibration was found to reduce clinically relevant junction errors on both units, from a maximum of 1.5 mm to 0.3 mm on Unit A and 1.2 mm to 0.7 mm on Unit B. The sum of cold junctions was reduced from 9.5 mm to 0.8 mm and from 4.9 to 0.6 mm, respectively. Mean jaw positional errors decreased significantly for some jaws and remained constant for others. Predicted jaw positions following calibration agreed well with measurements.

**Conclusions:**

Our results demonstrate the viability of this jaw calibration optimization program as a quality assurance tool. This program has been made open source and provides a unique QA solution for a common issue in centers operating linear accelerators for radiotherapy.

## 1 INTRODUCTION

The positional accuracy of linac jaws is assessed regularly for quality assurance, as recommended by the Canadian Partnership for Quality Radiotherapy (CPQR)^1^ and AAPM's TG‐142 report.^2^ Asymmetric and symmetric jaw settings are typically measured by projecting the source‐simulating field light of a linear accelerator onto graph paper or some other measurement surface, along the plane of the isocenter. Sub‐millimeter accuracy of jaw positions is generally required, being especially important for small‐field procedures such as stereotactic radiosurgery,^3^ where positional inaccuracies in the beam penumbra can greatly impact target dose levels. Positional errors in jaws that are used in clinical junctions are also of particular concern.

To mitigate positional inaccuracies, Varian TrueBeam (Varian Medical Systems, Palo Alto, CA, USA) users can calibrate the jaw positions in advanced service mode using a simple two‐point calibration procedure. This procedure involves setting each jaw to 1 cm and then 19 cm using the hand‐pendant on the couch. Afterwards, the jaw automatically moves to the mid‐range position (5 cm for Y‐jaws, and 9 cm for X‐jaws) so the user can verify the mid‐range positional accuracy before saving the calibration. This calibration must be performed with the gantry at 0 degrees. As mentioned by Corns and Zhao,^4^ this procedure does not allow the effects of collimator walkout or gantry sag^5^ to be accounted for during the calibration. It is, however, important for these effects to be considered, as even small inaccuracies in jaw positions can result in dosimetric errors of up to 15 %.^6^

In their 2019 article,^4^ Corns and Zhao implemented a method of setting jaw calibration positions so that the effects of collimator walkout can be considered during jaw calibration. This allows positional errors of the jaws to be optimized across multiple collimator angle settings, rather than at 0 degrees only. This method employs two steps: first, a mathematical model is used to determine the optimal jaw calibration origin, then the jaw positions are set to the appropriate position for calibration using their encoder values.

In this study, we expand upon their methodology^4^ to optimize the calibration over customizable combinations of gantry and collimator angles. Our methodology involves collecting Megavoltage Electronic Portal Imaging Device (MV EPID) images at a user‐defined set of gantry and collimator angles, and then running a script to optimize the absolute jaw positions while controlling the junction errors for clinically relevant junctions. The accuracy of jaw positions at certain gantry and collimator angles are particularly important, such as those that define junctions used in four‐field breast and craniospinal treatments. A weighted parameter is used in the optimization cost function to control the trade‐off between minimizing either the overall average jaw positional error or minimizing the error of jaws at certain junction positions. The optimization procedure also measures light‐field radiation field coincidence (LRFC), ensuring that this stays within tolerance. This feature requires the user to take LRFC MV images with the Standard Imaging FC‐2 phantom,^7^ but can be disabled if this is not available.

This methodology was tested locally on two Varian TrueBeam accelerators where certain junctions were out of tolerance (> 1 mm junction gap).

## 2 METHOD

The jaw calibration methodology consists of four stages: image collection, optimization, calibration, and verification. A procedure has been made available online, along with the open‐source JawCal code.^8^ When following along with the methodology, it is important to follow the precise folder and file naming conventions required for proper execution of the program.

### 2.1 Image collection

The purpose of image collection was to characterize the jaw positions at a variety of gantry and collimator angles. These images were analyzed by the JawCal program and used to determine the optimal calibration origins for each jaw. All images collected were 6X MV EPID images.

#### 2.1.1 Jaw encoder‐position correlation images

Corns and Zhao`s methodology^4^ of correlating jaw positions with encoder values was used to precisely set jaw positions. First, to mark the isocenter position in an image, Varian's isocenter cube phantom was placed directly on the EPID cover, as in Figure 1. The vertical position of the detector panel was set such that the cube was aligned at the isocenter. The vertical position of the detector was recorded, as it is required by the optimization program to accurately scale images and calculate jaw positions. The next step was to acquire a set of MV images with the gantry and collimator at 0 degrees and each jaw at a set of different positions (−0.5, 0, 0.5, 1, 1.5, 2, 5, 10, 15, 17, 18, 18.5, 19, 19.5, 20 cm). For each position of each jaw, the primary encoder value was noted from within service mode (Utilities—Encoder Display) and recorded.

**FIGURE 1:**
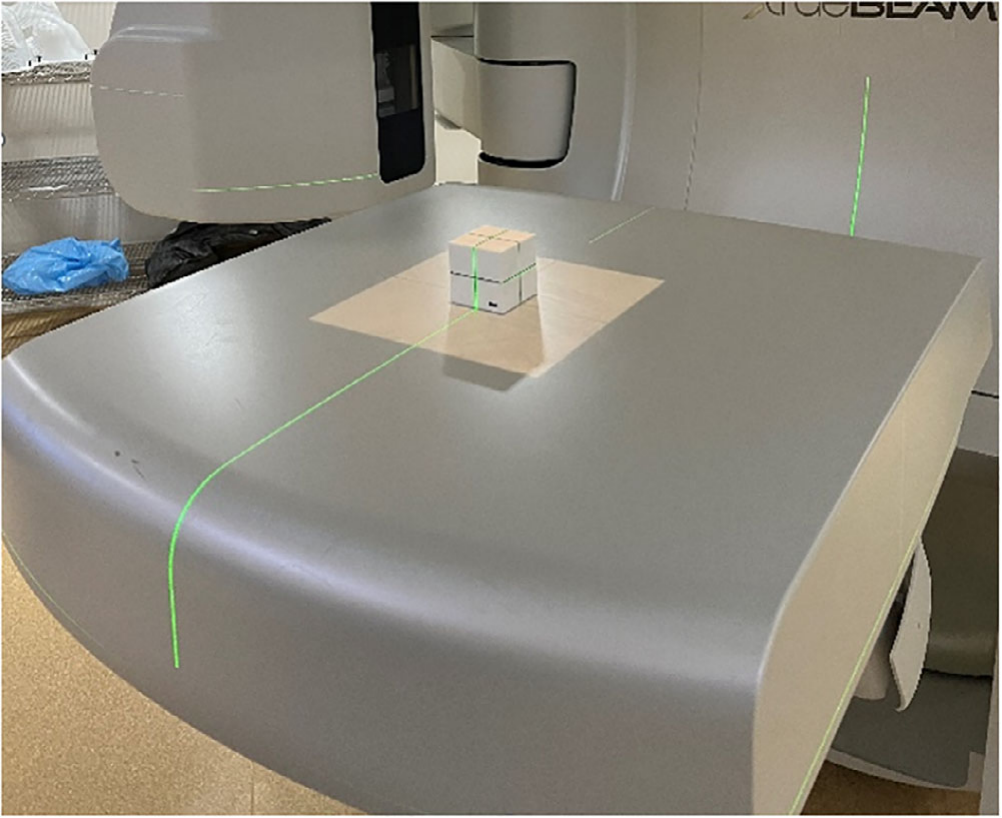
Varian's isocenter cube was placed on the EPID and aligned to the isocenter using the gantry's light field and the in‐room lasers.

#### 2.1.2 Jaw position‐assessment images

The next set of images to collect are used by the JawCal program to assess the machine`s jaw positions. There is no limit to the number of images that can be acquired for optimization. The goal was to construct an image set that was representative of the gantry sag and collimator walkout of each jaw. At a minimum, this should include gantry angles 0, 90, 180 and 270 degrees, with a range of collimator angles spanning those used clinically (e.g. 0, 90, 270 degrees).

The JawCal script can accept images collected at any gantry angle, while collimator angles are currently limited to 0, 90 and 270 degrees. At each gantry and collimator angle combination, four images are acquired (one for each jaw). Each image has the one jaw of interest set to a position of 0 cm, while all other jaws are at 10 cm. Jaws were moved to various positions at the control console in advanced service mode. It is important to note that jaw positions are determined relative to the isocenter location marked by the metal BB, and not by a single pixel on the EPID. This compensates for the effects of EPID sag,^9^ which would otherwise have an unknown affect on the calibration results.

For the program to assess the positional error of the closed jaw, the position of the isocenter must be indicated using Varian's isocenter cube phantom on the couch at each tested gantry angle (Figure 2). It was important that the cube phantom was removed from the field of view before acquiring half‐beam blocked jaw–assessment images. To minimize uncertainty and time associated with realigning the block at each gantry angle, the couch was moved longitudinally in and out of the treatment field to the same precise location at each gantry angle. To avoid having the phantom slide out of place, bolus was placed under the block for added friction.

**FIGURE 2:**
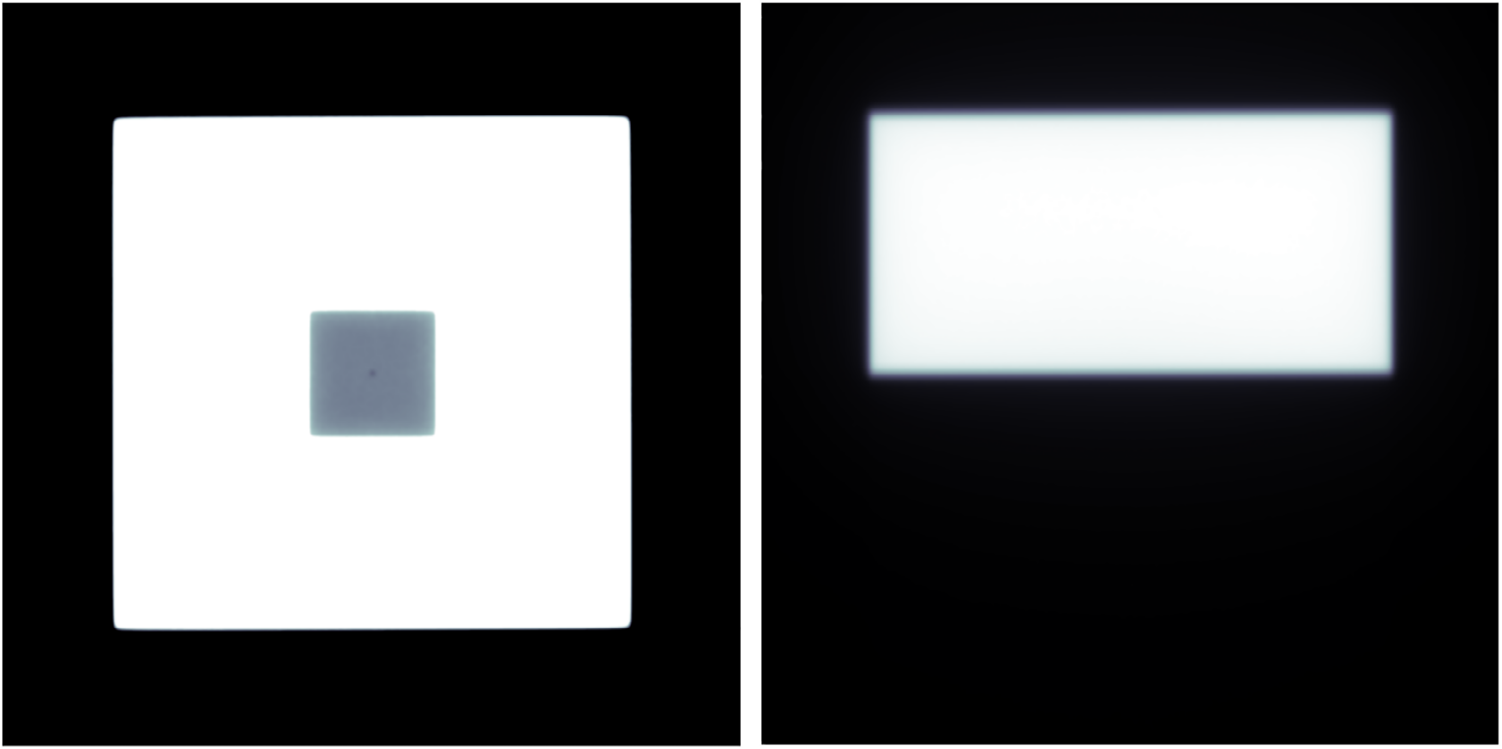
At each gantry angle, the isocenter cube is brought back into the field of view by moving the couch longitudinally. An image of the block is collected (left), as well as all closed‐jaw images needed for characterization of jaw errors (right).

For our clinical calibration, the image set used for testing this procedure is represented in Table 1. These angles were chosen as they represent the patterns of errors across the full range of gantry and collimator positions, without being overly excessive from a time‐cost perspective. Furthermore, the gantry angles of 0, 50, 130, 180, 230 and 310 degrees with the collimator at 90 degrees correspond to positions used locally to match jaws at 4‐field breast junctions. After all half‐beam blocked images were acquired, the next step was to acquire LRFC images with the Standard Imaging FC‐2 phantom.^7^ This can be performed with 6 MV, using 10 and 15 cm fields.

**TABLE 1:** The gantry and collimator positions for each jaw used in optimization. Jaw positions used in clinically relevant junctions are listed in the third column. Four images are collected at each gantry and collimator angle combination (one for each jaw).

Gantry Angle (Degrees)	Collimator Angle (Degrees)	Jaws Used in Clinical Junctions
0	0, 90, 270	X1
50	0, 90, 270	X2
90	0, 90, 270	None
130	0, 90, 270	X2
180	0, 90, 270	X1
230	0, 90, 270	X2
270	0, 90, 270	None
310	0, 90, 270	X2

Finally, for quality assurance purposes, open field MV images were acquired with four symmetric jaw positions (5 cm, 10 cm, 15 cm, 20 cm). These images are not used for optimization, but are analyzed automatically and output in the results spreadsheet as the current jaw positions. This was repeated after calibration for comparative purposes.

All images were then exported as DICOM files from the image workspace in service mode, and files and folders were named according to specific conventions which must be adhered to for the optimization program to execute properly.

### 2.2 Optimization

The next step was to run the JawCal script to produce a results spreadsheet which contains information including current jaw positions and errors, LRFC results, errors in 4‐field breast junctions, and predicted values following calibration. Note that clinical junctions can be modified/added/removed in the code to correspond to local clinical practices.

In addition to generating the results spreadsheet, the program also produces an image which plots the current jaw‐encoder relationship. While Corns and Zhao^4^ fit a cubic polynomial function to the data, we took a slightly different approach by fitting three separate cubic functions to different regions of jaw positions, as in Figure 3. This was done to improve the fit in each of these regions separately. Note that jaw positions are defined in the penumbra as the center of the pixel having a signal closest to 50 % of the open field dose. All images were first supersampled to triple resolution in each dimension with cubic interpolation (producing nine times the original pixels), and the open field dose was approximated as the median of the 20,000 pixels of highest signal in the post‐supersampled image.

**FIGURE 3:**
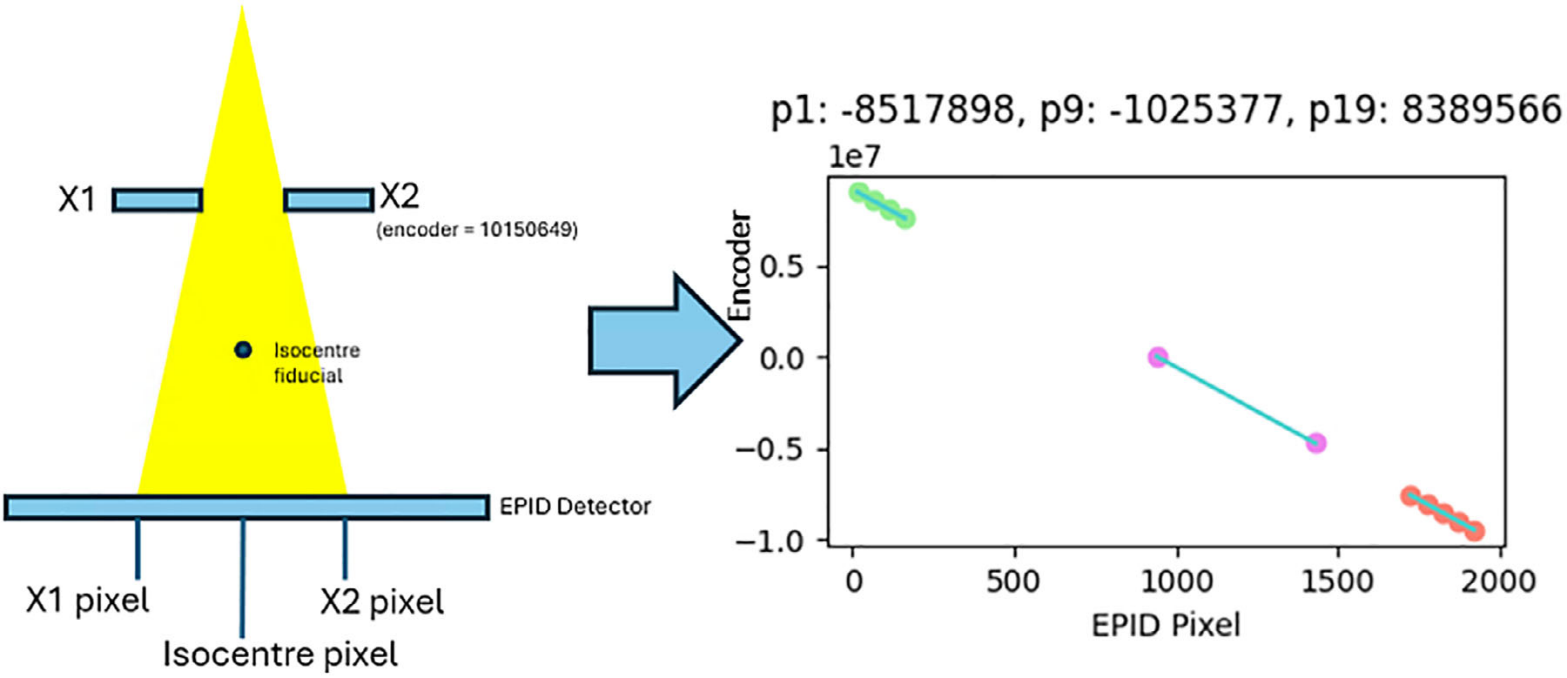
The encoder–EPID pixel relationship is defined by fitting separate cubic polynomial functions to regions of low and high jaw position values. After the JawCal program computes the optimal origin for each jaw's calibration, which is defined in terms of EPID pixels, these curves are used to obtain encoder values which can be used to accurately set the jaws during calibration. Jaw positions are in millimeters and p1, p5, p9, and p19 define the encoder values at jaw positions of 1, 5, 9, and 19 cm, respectively.

After JawCal processes all jaw positions and errors and completes the LRFC analysis, an optimization cost function, C, was used to determine the optimal calibration origins for each jaw. The form of C is given in Equation 1.

(1) $$C=\frac{1-j}{N}\sum_{i=1}^{N}Jaw_{i}+\frac{j}{8}\sum_{i=1}^{8}\left(junction_{i}+CJ_{i}\right)+LRFC$$

j is a customizable parameter used to control the relative importance of junction errors over total mean error during optimization. The default value was set to 0.7, as the clinical impact of uncertainty in the penumbra position was particularly concerning at clinical junctions, and 0.7 was found to provide a reasonable trade‐off between junction and mean jaw positional errors following calibration. However, a robust experimentation with the setting of j was not performed. N is the total number of half‐beam blocked jaw images acquired, and $Jaw_{i}$ is the positional error of the i ^th^ jaw, and i is summed over N. Similarly, $junction_{i}$ is the total positional error of the i^th^ junction, and is summed over all clinical junctions (8 in our case). The convention is such that a positive junction indicates a hot junction, and a negative junction is a cold junction, as illustrated in Figure 4.

**FIGURE 4:**
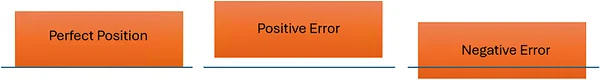
The sign convention for jaw positions is illustrated. The convention is defined along the axis of travel of each jaw, so there is no one set directional axis with respect to the linac.

While both positive and negative junction errors are undesirable, it is more detrimental to have cold junctions in 4‐field breast plans, as insufficient dose below the junction could compromise target coverage. Therefore, an additional junction parameter, $CJ_{i}$ (the i^th^ cold junction) was added to the cost function. This was defined such that it is 0 if a junction's error was non‐negative, or its absolute value if it is negative.

The LRFC term was defined as the average displacement of the radiation field in each dimension from the light field crosshair, and is averaged over all LRFC images obtained. If the displacement of any dimension is less than 0.4 mm, it was zeroed. If it is greater than 0.7 mm, it was weighted by an extra factor of three. This was done to avoid any LRFC tolerance issues which negate the efficacy of the calibration.

To find the optimal origin for jaw calibration, the program iterates over a 4 × 4 grid of X1, X2, Y1, and Y2 jaw positions. The range of each jaw tested is half a millimeter from either side of the isocenter. 31 and 21 evenly spaced positions are tested for X and Y jaws, respectively. The X jaws were sampled with higher resolution than Y jaws, as only X jaws are used to form local clinical junctions, and thus finer precision was desired in the X direction. In this way a total of 423,801 possibilities were assessed.

After the program has computed the total cost for each point in the 4 × 4 grid, the point corresponding to the minimal cost defines the jaw positions to be used during calibration. The next step of the program was to translate these positions, which are described in terms of EPID pixels, into encoder values. This was done using cubic polynomial fits of encoder–pixel relationships (Figure 3).

Once the program has finished, the calibration can be performed, following the procedure described in detail by Corns and Zhao.^4^ This involves initiating the jaw calibration for each jaw in advanced service mode, and setting jaw positions with the couch hand pendant. Unfortunately, as the sensitivity of the pendant is high, some back‐and‐forth iteration of positioning was required before falling “close enough” (arbitrarily within 500) to the optimal calibration encoder value.

Following each calibration, the jaw automatically moves to the middle of its range of motion (9 cm for Y jaws, 5 cm for X jaws), and the encoder value of this position was compared to the value predicted by the program. This was found to be within 5000, which corresponds to approximately 5 % of a millimeter.

### 2.3 Quality assurance

The next step was to verify the results of the calibration. This was done by first repeating all stages of the image collection phase, and re‐running JawCal. This was useful for seeing whether post‐calibration measurements were close to those predicted by the program. We also performed asymmetric jaw positional QA on graph paper, a machine performance test (Varian's MPC test), and our local jaw‐matching QA procedure, which uses an in‐house phantom.

### 2.4 Local clinical testing

This procedure was evaluated on two TrueBeam units, Unit A and B. Average and maximum jaw and junction errors were compared before and after calibration. Junction offsets or errors were obtained by appropriately summing the jaw errors of the matched jaws composing the junction. Furthermore, to characterize the particularly detrimental effect of having “cold junctions” in 4‐field breast treatments, where jaw errors cause a region of low dose in the target volume, we recorded the sum of cold junctions (SCJ) before and after calibration. This simply sums over the positional errors of all cold junctions measured, such that “Hot” jaws do not contribute to this metric. A high SCJ measurement is undesirable.

Finally, the significance of parameter changes following calibration was assessed using a paired t‐test.

## 3 RESULTS

Mean parameters measured before and after calibration are compared in Table 2, along with significance values for changes. Figure 5 illustrates how junction errors significantly improved following calibration, and Figure 6 compares predicted post‐calibration junction errors with actual errors measured. Junction errors were brought below tolerance following calibration on both units. Changes in maximum jaw and junction values, as well as changes in the SCJ, are reported in Table 3. A heat map of the cost function for various X and Y jaw positions is shown in Figure 7. The sum of cold junctions was significantly diminished following calibration, as shown in Figure 8.

**TABLE 2:** Mean jaw and junction positional errors before and after calibration, along with the significance in their changes are shown for each TrueBeam unit (A and B).

		Mean jaw position error (mm)		
	Jaw	Before calibration	After calibration	*p*‐value
**Unit A**	**X1**	0.8±0.1	0.4±0.2	< 0.01
	**X2**	0.4±0.2	0.4±0.2	1
	**Y1**	0.4±0.2	0.4±0.2	1
	**Y2**	0.4±0.2	0.4±0.3	1
	**Junctions**	1.2±0.2	0.2±0.2	< 0.01
**Unit B**	**X1**	0.2±0.1	0.2±0.2	1
	**X2**	0.6±0.3	0.3±0.1	< 0.01
	**Y1**	0.7±0.3	0.2±0.1	< 0.01
	**Y2**	0.3±0.2	0.3±0.3	1
	**Junctions**	0.6±0.2	0.4±0.2	< 0.01

**FIGURE 5:**
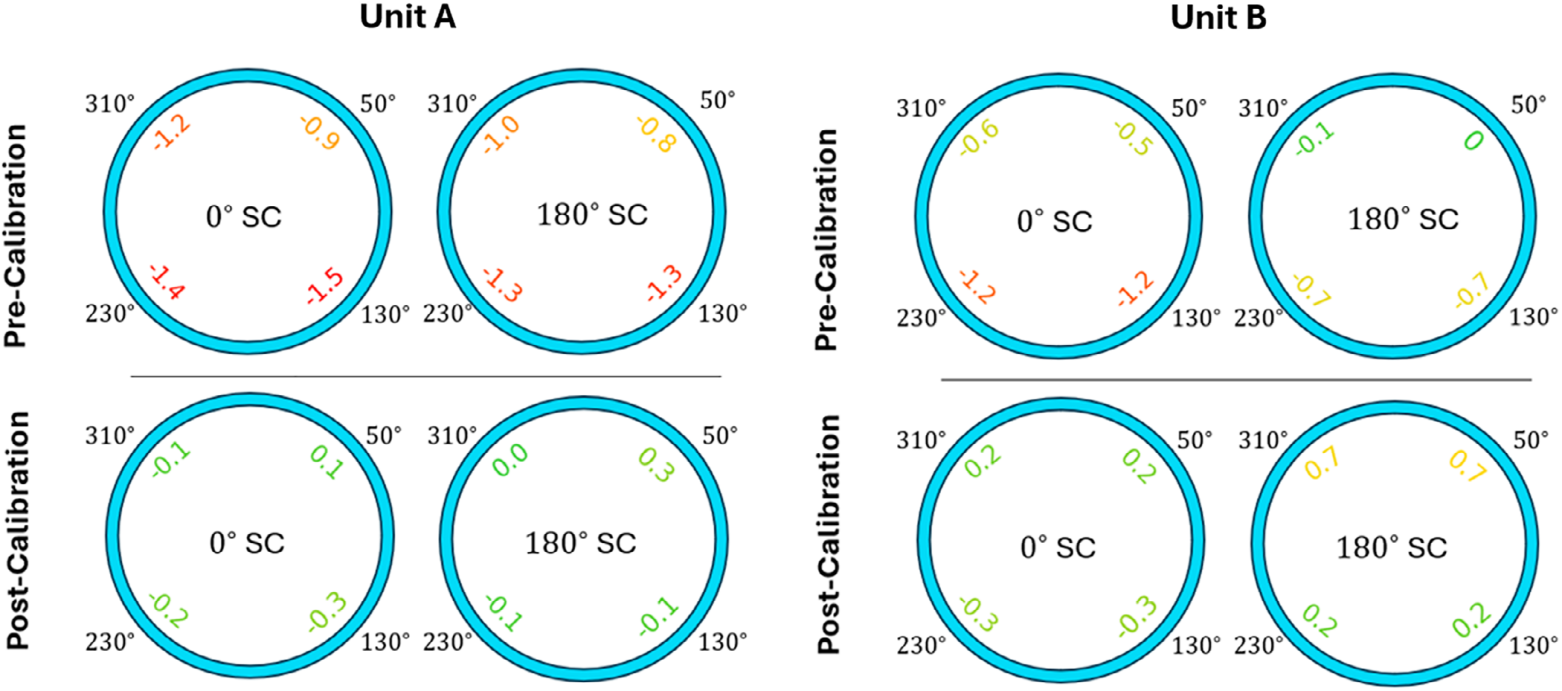
The effect of the calibration procedure on the clinically relevant junctions (top) and average absolute jaw errors (bottom) is illustrated for each of the two units tested (A and B). Junctions were tested between tangent fields (gantry angles of 50, 130, 230, and 310 degrees) and supraclavicular fields (at 0 and 180 degrees). Each blue circle shows the junctions between a given supraclavicular field's X1 jaw and the X2 jaw of tangent fields at 4 different gantry angles.

**FIGURE 6:**
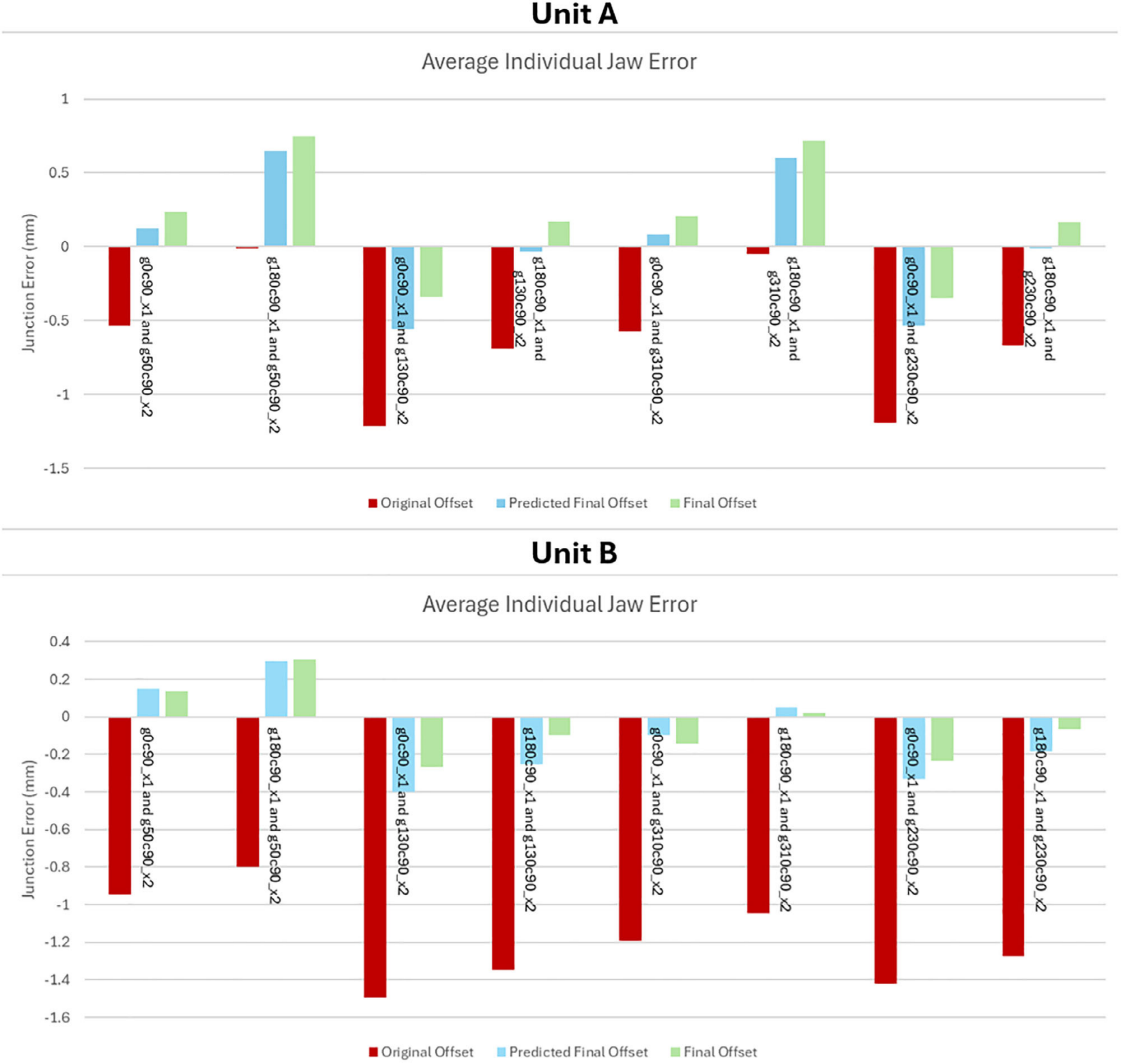
For each clinical junction included in the optimization of jaw calibration positions, the original junction error, predicted error following calibration, and measured value following calibration are shown. Note that negative values correspond to “cold” junctions, which was penalized during optimization as described in the methodology. As an example for understanding the labels, g0c90_x1 and g50c90_x2 describes the junction formed between the x1 jaw at a gantry angle of 0 degrees and collimator angle of 90 degrees, and the x2 jaw at a gantry angle of 50 degrees and a collimator angle of 90 degrees.

**TABLE 3:** Maximum jaw and junction positional errors before and after calibration, along with the significance in their changes are shown for each TrueBeam unit (A and B).

Unit	Before calibration (mm)	After calibration (mm)
	**Max junction error**	
**A**	1.5	0.3
**B**	1.2	0.7
	**Max jaw error**	
**A**	1	0.9
**B**	1.1	0.8
	**Sum of cold junctions (SCJ)**	
**A**	9.5	0.8
**B**	4.9	0.6

**FIGURE 7:**
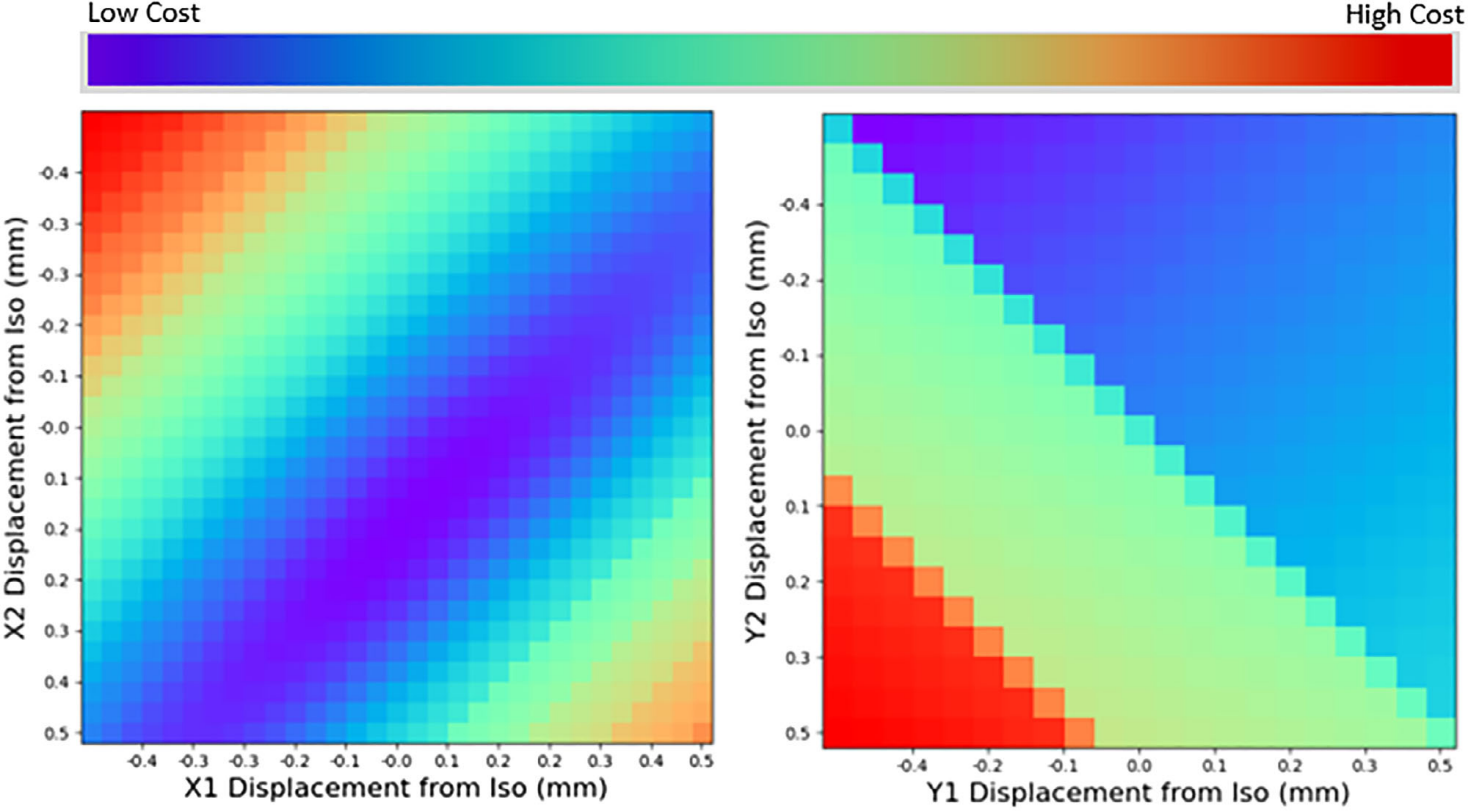
An example of the cost function along two separate two‐dimensional slices of the grid‐search matrix. Note that purple indicates the lowest cost, while red indicates the highest. The pattern seen in the left image, for the X‐jaws, exists as the junction error is optimal along a diagonal line. The pattern in the right image is due to the LRFC value becoming within tolerance, and then in action, towards the bottom left of the grid slice.

**FIGURE 8:**
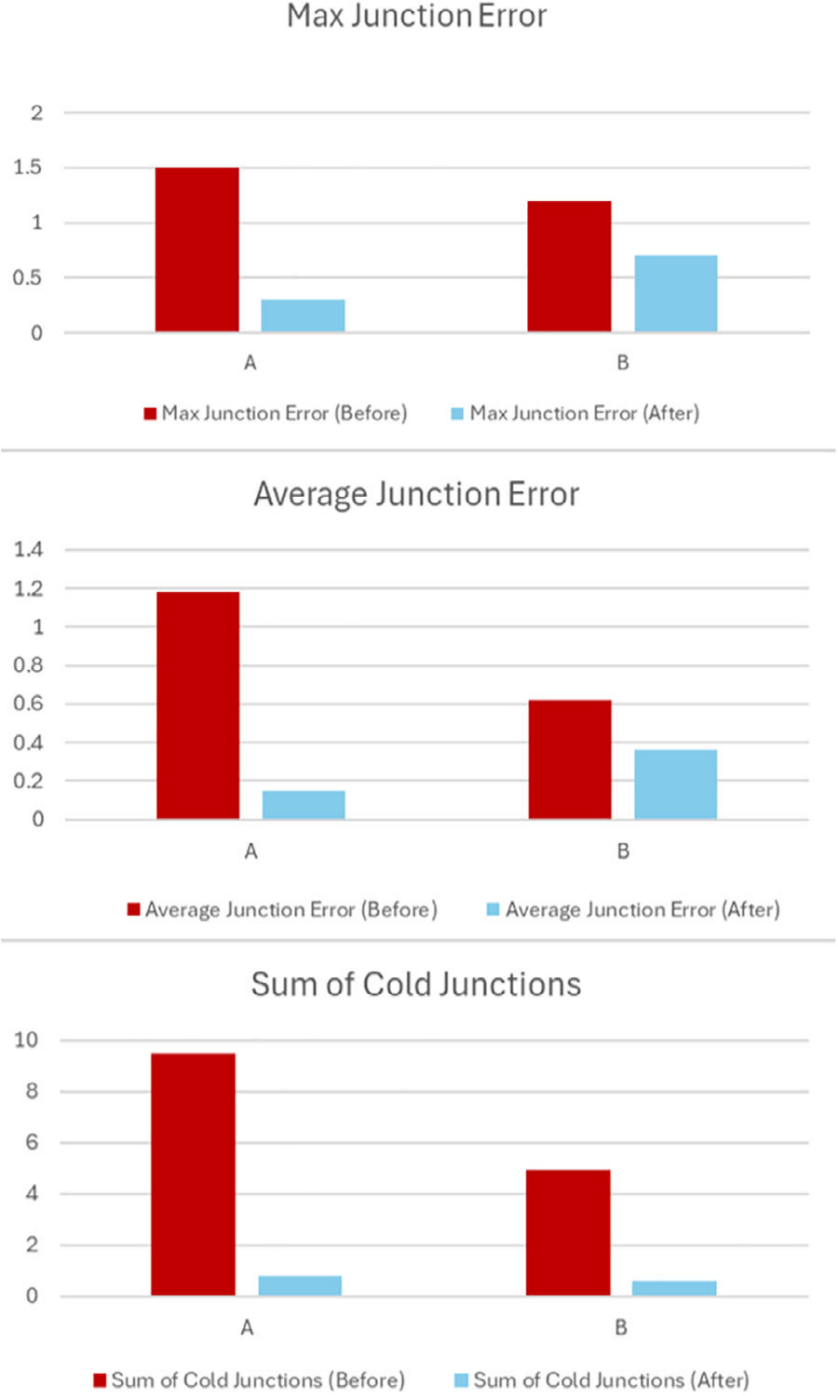
Mean and maximum junction errors (over 8 clinical junctions tested) are shown, along with the sum of cold junctions (SCJ) before and after calibration.

## 4 DISCUSSION

The results of this study validate the outlined methodology as a viable tool for correcting jaw and junction positional errors on Varian TrueBeam accelerators. Prior to jaw calibration, both units tested, A and B, had junction errors that were out of tolerance (> 1 mm). For both, the jaw calibration brought the junctions within tolerance while reducing the average jaw positional error and ensuring LRFC stayed within tolerance. Notably, this was achieved while also reducing the SCJ significantly, as seen in Figures 6 and 8. The close agreement between predicted and measured jaw and junction errors seen in Figure 6 validates the ability of encoder calibration methodology.

This work builds off the 2019 framework developed by Corns and Zhao,^4^ which used affine transformations and MV EPID imaging to optimize jaw origins based on collimator rotations. The modifications to the methodology implemented in this work allow the jaw calibration to be optimized over gantry angles and allows for greater flexibility of desired results. For instance, by setting the parameter, j, in the cost function, one can control the weighting of junction and jaw errors during optimization. Furthermore, the procedure allows for images to be collected and analyzed automatically at any gantry angle.

As seen in Table 3, the maximum jaw error on Unit B, while brought below the action threshold of 1 mm, was still 0.8 mm following calibration. This may be improved by adjusting the junction priority factor in the cost function, but this methodology is limited to splitting the difference of jaw offset values at various gantry/collimator angles, and it is not a direct fix to gantry and collimator sag. In this case, the accuracy of junctions was prioritized over the positional accuracy of individual jaws at each gantry angle.

Some technical challenges with this approach remain. Setting jaws to the precise calibration location is tedious using the hand pendant. However, no alternative exists, and a high degree of accuracy is still achievable with this method. This limitation only necessitates some iterative aligning of the jaws during calibration.

Another limitation of the methodology is that it assumes that the isocenter fiducial is perfectly aligned at the mechanical isocenter. It is therefore imperative that great care is taken during this alignment step. The method also assumes that the imager is perfectly aligned orthogonally with the radiation beam, and that the vertical position of the EPID displayed on the monitor is precisely the distance between the source and the detector. Moving the couch longitudinally in and out of the isocenter position also could create additional uncertainty; however, we found that the position was highly reproducible using this approach. The finite pixel size of the EPID (approximately 0.3 mm) adds an additional source of uncertainty to this methodology.

Uncertainty related to jaw position reproducibility was not quantified in this work; however, we found that repeating the procedure produced nearly identical results. Jaw reproducibility has been found to be within less than 0.2 mm on Varian TrueBeam machines (less than the EPID pixel size).^10^ Similarly, the uncertainty of moving the couch in and out of the field of view between measurements remains unquantified here, but is specified by Varian to be less than 0.5 mm).

The validation of this work was limited to two Varian TrueBeam machines, and this methodology would benefit from validation from other centers. Due to time and resources, a robust analysis of the impact of various settings of the parameter j in the cost function was not performed; however, it is possible that other settings of j could strengthen the results of the calibration. Further work on the design of the cost function would be a valuable next step for future work.

As previously mentioned, small inaccuracies in jaw positions can result in dosimetric errors of up to 15 %,^6^ and junction matching is particularly important in 4 field breast and 3D conformal craniospinal irradiation. To achieve 5 % tolerance and 10 % action levels for the dose peak/valley in field junctions requires jaw accuracy of less than half a millimeter.^11^ Implementing this jaw calibration procedure allows jaw errors to be minimized across the entire range of gantry and collimator angles, rather than just at 0 degrees. This minimizes dose variation over gantry/collimator angles that are often overlooked in routine clinical quality assurance.

The results of this work indicate that large errors can exist in jaw positions at different gantry angles, even if the jaw positional quality assurance at a gantry angle of 0 degrees is within tolerance. This methodology and JawCal code have been made open source^8^ for others to implement and build upon.

## 5 CONCLUSION

The jaw calibration optimization methodology presented here offers a robust solution for correcting jaw positional errors caused by both gantry sag and collimator walkout. By implementing a weighted cost function, our results demonstrate that jaws on TrueBeam units can be calibrated to achieve sub‐millimeter junction accuracy across a range of customizable angles.

## AUTHORS CONTRIBUTION

Caleb Sample, Marcus Sonier and Parmveer Atwal were involved in the design of the methodology. Code development and testing of the methodology was executed by Caleb Sample. The manuscript was drafted by Caleb Sample and revisions were made by Marcus Sonier and Parmveer Atwal.

## CONFLICT OF INTEREST STATEMENT

The authors have no relevant conflicts of interest to disclose.

## FUNDING STATEMENT

This study was not supported by any external sources of funding.

## ETHICS STATEMENT

As this study involved no human participants, research ethics board approval was not necessary.

## Data Availability

The repository containing the code used in our methodology as well as a detailed procedure are made available on our cited Github link.
